# Risk Factors and Treatment Effectiveness in the Recovery and Recurrence of Plantar Fasciitis: A Single-Center Study at King Saud Medical City, Riyadh, Saudi Arabia

**DOI:** 10.7759/cureus.112265

**Published:** 2026-07-08

**Authors:** Yasser S Aljaied, Mohamed Alrubaiaan, Mazin Elsarrag, Bandar ALshehry, Rahaf Almutairi, Ghada F AlSwaji, Abdulmlk A Alqasem, Hamza Enabi

**Affiliations:** 1 Family Medicine, King Saud Medical City, Riyadh, SAU; 2 Medicine, Alfaisal University, Riyadh, SAU; 3 Medicine, Imam Mohammad Ibn Saud Islamic University, Riyadh, SAU; 4 General Practice, Meena Clinics, Riyadh, SAU

**Keywords:** heel pain, king saud medical city, pain severity, plantar fasciitis, recurrence, saudi arabia

## Abstract

Background: Plantar fasciitis is a frequent cause of heel pain, and local evidence describing risk factors, recurrence patterns, and treatment outcomes in Saudi Arabia remains limited.

Objective: The objective of the study is to investigate the epidemiology, risk factors, treatment types and outcomes, recurrence rates, and recovery patterns of plantar fasciitis at King Saud Medical City in Riyadh, Saudi Arabia.

Methods: A retrospective cohort study was conducted at King Saud Medical City in Riyadh, Saudi Arabia, from May 1, 2023, to November 28, 2025. Due to limited follow-up documentation in routine hospital records, data were collected using structured telephone interviews, while the hospital electronic medical record system was used to confirm eligibility by verifying the diagnosis of plantar fasciitis and to obtain updated contact information; verbal consent was obtained prior to interviews. A total of 121 adult patients were identified; 10 were included in a pilot phase and excluded, leaving 111 eligible patients, of whom 93 completed interviews and were included in the final dataset. Responses were entered into a structured electronic form during interviews and exported for cleaning and verification prior to analysis. Categorical variables were summarized using frequencies and percentages, and ordinal outcomes were summarized using medians and interquartile ranges; associations were evaluated using the Chi-squared test, with statistical significance set at a probability value less than 0.05.

Results: Among 93 participants, the largest age group was 51 to 60 years (29 (31.18%)), and women comprised 51 (54.84%). Sedentary occupation (73 (78.49%)) and physical inactivity based on World Health Organization recommendations (69 (74.19%)) were common. Most participants reported a single episode over the previous two years (76 (81.72%)), while recurrence occurred in 17 (18.28%). Symptom duration in the first episode was predominantly acute (72 (77.40%)), with fewer subacute (17 (18.30%)) and chronic (4 (4.30%)) presentations; recurrent episodes showed a shift toward longer subacute courses without chronicity. Management remained predominantly conservative across episodes, with rest or activity modification and non-steroidal anti-inflammatory drugs used in every reported episode; during the first episode, stretching exercises (68 (73.10%)), avoidance of non-supportive footwear (63 (67.70%)), and external support (58 (62.40%)) were commonly reported. Pain severity improved after treatment across episodes, with median pain scores decreasing from 5.00 to 2.00 in the first episode, 5.00 to 1.00 in the second episode, 5.00 to 1.00 in the third episode, and 4.00 to 3.00 in the fourth episode. Satisfaction was high, with 52 (55.91%) very satisfied and 25 (26.88%) satisfied. Recurrence was significantly associated with obesity, with a Chi-squared value of 4.25 and a probability value of 0.039; recurrence was more frequent among obese than non-obese participants, 28.57% versus 9.80%.

Conclusion: In our cohort from King Saud Medical City, plantar fasciitis was managed mainly with conservative measures and was associated with meaningful patient-reported pain improvement and high satisfaction; however, recurrence was not uncommon and was significantly associated with obesity, supporting the importance of addressing weight-related risk alongside conservative management to reduce recurrence.

## Introduction

Plantar fasciitis is one of the most common causes of heel pain, affecting both athletes and non-athletes, and approximately one in 10 people will experience it during their lifetime [1]. Its peak incidence occurs between 40 and 60 years of age, and it affects women more frequently than men [1]. Although the exact etiology remains unclear, plantar fasciitis is believed to be an overuse-related condition characterized by degenerative rather than inflammatory changes in the plantar fascia, leading some authors to suggest the terms fasciosis or fasciopathy [1].

Several modifiable and structural risk factors have been associated with plantar fasciitis. Modifiable factors include repetitive high-impact activity, obesity (body mass index (BMI) > 27 kg/m²), prolonged weight-bearing, sedentary lifestyle, and intrinsic foot and/or calf muscle tightness. Structural factors include limited ankle dorsiflexion, pes planus, pes cavus, and leg length discrepancy (LLD) [1]. Clinically, plantar fasciitis typically presents as gradual-onset stabbing heel or sole pain over the medioplantar surface, usually worse with the first steps in the morning or after inactivity, improving with movement but recurring or worsening after prolonged weight-bearing. Examination commonly reveals tenderness at the calcaneal insertion of the plantar aponeurosis [1]. Diagnosis is primarily clinical; the Windlass test may aid assessment, while imaging is generally reserved for unclear cases or persistent pain [2].

Management is usually conservative and may include rest, avoidance of aggravating factors, pressure reduction using heel inserts, stretching exercises, shoe inserts, orthotic support, physical therapy, non-steroidal anti-inflammatory drugs (NSAIDs), and corticosteroid injections in selected cases. In persistent or chronic cases, additional interventions such as extracorporeal shockwave therapy (ESWT) or surgery may be considered [2]. However, despite the available treatment options, there is no universally accepted gold-standard treatment. Recurrence and persistent symptoms remain important clinical concerns. Therefore, identifying factors associated with plantar fasciitis development and recurrence, as well as evaluating treatment effectiveness, may help guide clinical decision-making.

Plantar fasciitis is a leading cause of heel pain, yet local evidence describing its clinical burden and real-world management in Saudi Arabia remains limited. This gap leaves clinicians without clear local benchmarks for typical presentation, symptom duration, treatment patterns, and recurrence risk.

To address this gap, we conducted a retrospective cohort study at King Saud Medical City, a tertiary care center within the First Health Cluster in Riyadh, Saudi Arabia. We aimed to describe the demographic and clinical profile of adults with plantar fasciitis, examine episode patterns and symptom duration over two years, estimate recurrence, describe conservative management strategies across first and recurrent episodes, evaluate patient-reported outcomes, and assess factors associated with recurrence, including BMI and other patient characteristics.

## Materials and methods

Study design and setting

This retrospective cohort study was conducted at King Saud Medical City in Riyadh, Saudi Arabia, a major tertiary care facility under the First Health Cluster. The study was designed to investigate key clinical and patient-reported dimensions of plantar fasciitis, including associated risk factors, treatment modalities, recurrence patterns, and recovery outcomes. The study period spanned from May 1, 2023, to November 28, 2025.

This study was reviewed and approved by the Institutional Review Board at King Saud Medical City, Riyadh, Saudi Arabia (IRB Registration Number with KACST: H-01-R-053; U.S. Department of Health and Human Services IORG #: IORG0010374). The study was granted exempt approval (type of review: initial; category of approval: exempt) under Proposal Reference No. H1RI-09-Mar25-02, approved on March 12, 2025, with validity until March 11, 2026.

Due to the lack of consistent follow-up visits and limited documentation in routine hospital records, data collection was performed via structured telephone interviews. The hospital’s electronic medical record system, Watheeq, was used to confirm the eligibility of patients by verifying the diagnosis of plantar fasciitis and to obtain their most updated contact information.

Study population and sampling procedure

A total of 121 adult patients were identified in the medical record system as having a confirmed diagnosis of plantar fasciitis during the study period. From this group, a subset of 10 individuals was involved in a pilot phase designed to test and refine the study questionnaire. These patients were excluded from the final analysis to maintain methodological rigor and prevent duplication of data. This resulted in a final eligible sample of 111 patients.

Out of the 111 eligible individuals, 93 patients successfully completed the telephone interviews and were included in the final dataset. The remaining patients could not be reached or declined participation despite multiple telephone contact attempts conducted on different days and at various times of the day.

For this study, a recurrent episode was defined as a new occurrence of plantar fasciitis following a pain-free interval of at least three consecutive months. A single episode was defined as any episode of plantar fasciitis without a pain-free interval of three months or more. Episode duration was classified as acute, less than six weeks; subacute, 6-12 weeks; or chronic, more than 12 weeks.

Data collection instrument and procedure

The data collection tool, a structured questionnaire, was developed by the investigators in alignment with the study objectives. To ensure its clinical appropriateness and content validity, the instrument underwent expert review in orthopedics, family medicine, and physical medicine and rehabilitation. A pilot test was then conducted with 10 patients, whose feedback was used to optimize clarity, logical flow, and overall comprehension. These pilot cases were excluded from the final analysis. The finalized questionnaire encompassed five principal domains: demographic and lifestyle characteristics, underlying risk factors, episode-specific features, treatment modalities used, and patient-reported outcomes (Appendix).

Telephone interviews were conducted by all authors. Before data collection, the authors agreed on how to ask the questionnaire items and how to clarify questions to participants in a consistent manner. Responses were directly entered by the investigators into a structured Google Form (Google, Mountain View, CA, USA) during each interview. After data collection was completed, responses were exported into Microsoft Excel (Microsoft Corp., Redmond, WA, USA) for cleaning and verification. The investigators reviewed the dataset for completeness, accuracy, and consistency prior to statistical analysis.

Because data collection relied on telephone interviews, recall bias was considered a potential limitation, particularly for patient-reported episode history, symptom duration, and prior treatment use. To reduce non-response, eligible participants were contacted multiple times on different days and at different times of the day.

Statistical analysis

Data were analyzed using IBM SPSS Statistics for Windows, Version 29.0 (IBM Corp., Armonk, NY, USA). Categorical variables were summarized as frequencies and percentages, while ordinal variables were summarized using medians and interquartile ranges (IQRs). Associations between categorical variables, including recurrence in relation to participant characteristics and treatment categories, were assessed using the Chi-squared test. Changes in pain severity before and after treatment were described descriptively using medians and IQRs. All tests were two-tailed, and statistical significance was defined as a p-value less than 0.05. Only univariate Chi-squared analyses were performed because of the modest sample size and the low number of recurrence cases, which limited the suitability of multivariable modeling.

## Results

Among the 93 participants, the majority were older adults, with the highest representation in the 51-60 years age group, 29 (31.18%), followed by 41-50 years, 25 (26.88%), and 61-70 years, 22 (23.66%). There was a modest predominance of women, 51 (54.84%), compared with men, 42 (45.16%). Most participants had sedentary occupations, 73 (78.49%), and 69 (74.19%) were classified as physically inactive according to World Health Organization (WHO) criteria, as shown in Table 1.

**Table 1:** Characteristics of Participants (N = 93) Occupation was categorized based on the level of physical demand: sedentary jobs involve mostly sitting with minimal movement, active jobs require regular standing or walking, and highly active jobs involve sustained physical effort such as lifting or continuous motion. Physical activity level was classified according to World Health Organization (WHO) recommendations: physically inactive refers to engaging in less than 150 minutes of moderate activity per week or less than 75 minutes of vigorous activity, with no regular muscle-strengthening exercises. Active individuals meet the WHO minimum of 150-300 minutes of moderate activity or 75-150 minutes of vigorous activity per week, along with strength training on at least two days per week. Highly active individuals exceed these recommendations, performing more than 300 minutes of moderate activity or more than 150 minutes of vigorous activity per week, with consistent strength training.

Variable	Category	n (%)
Age	18-30 years	6 (6.45%)
	31-40 years	11 (11.83%)
	41-50 years	25 (26.88%)
	51-60 years	29 (31.18%)
	61-70 years	22 (23.66%)
	Total	93 (100%)
Gender	Male	42 (45.16%)
	Female	51 (54.84%)
	Total	93 (100%)
Occupation	Sedentary	73 (78.49%)
	Active	11 (11.83%)
	Highly active	9 (9.68%)
	Total	93 (100%)
Physical activity level	Physically inactive	69 (74.19%)
	Active	13 (13.98%)
	Highly active	11 (11.83%)
	Total	93 (100%)

**Figure 1:**
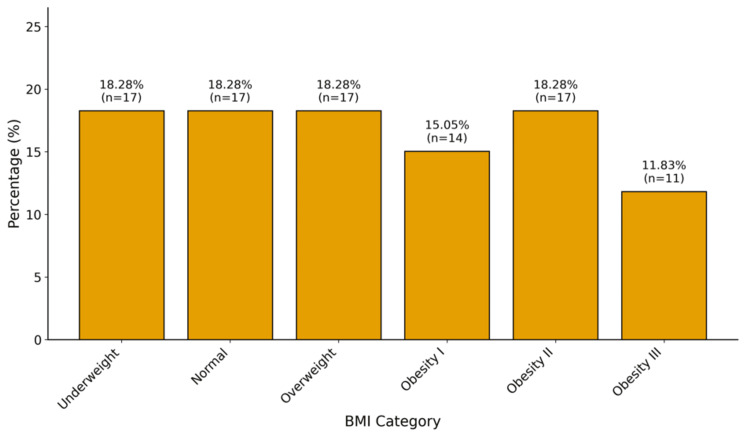
Body Mass Index (BMI) Categories Among Participants (N = 93) BMI categories were classified according to World Health Organization (WHO) standards: underweight (BMI < 18.5), normal weight (18.5-24.9), overweight (25-29.9), obesity Class I (30-34.9), obesity Class II (35-39.9), and obesity Class III (≥40). Percentages and absolute counts (n) are labeled for each category.

When we look at BMI categories, our study shows a broad spread across weight groups, rather than clustering in a single category. The distribution highlights that plantar fasciitis is encountered across the BMI spectrum, as shown in Figure 1.

**Figure 2:**
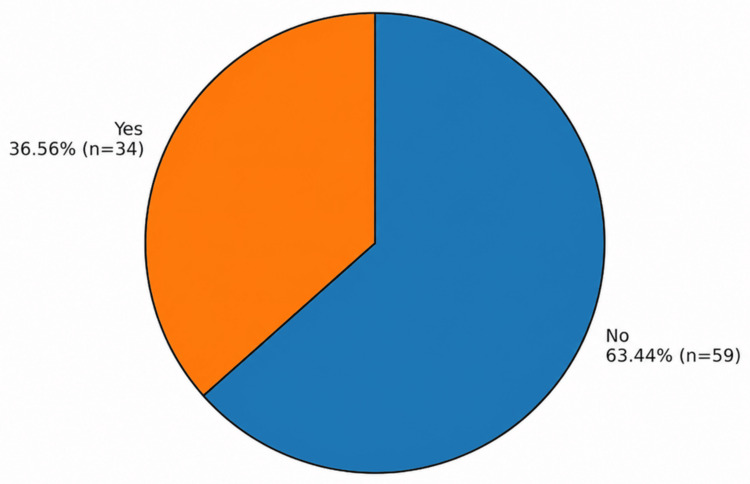
Smoking or Vaping Status Among Participants (N = 93) The figure presents the proportion of participants who reported current use of cigarettes or vapes. Percentages and absolute counts (n) are labeled for each category.

As shown in Figure 2, the majority of participants did not smoke or vape (59 (63.44%)). In contrast, 34 (36.56%) reported smoking or vaping, with a mean smoking exposure of 13 pack-years.

**Figure 3:**
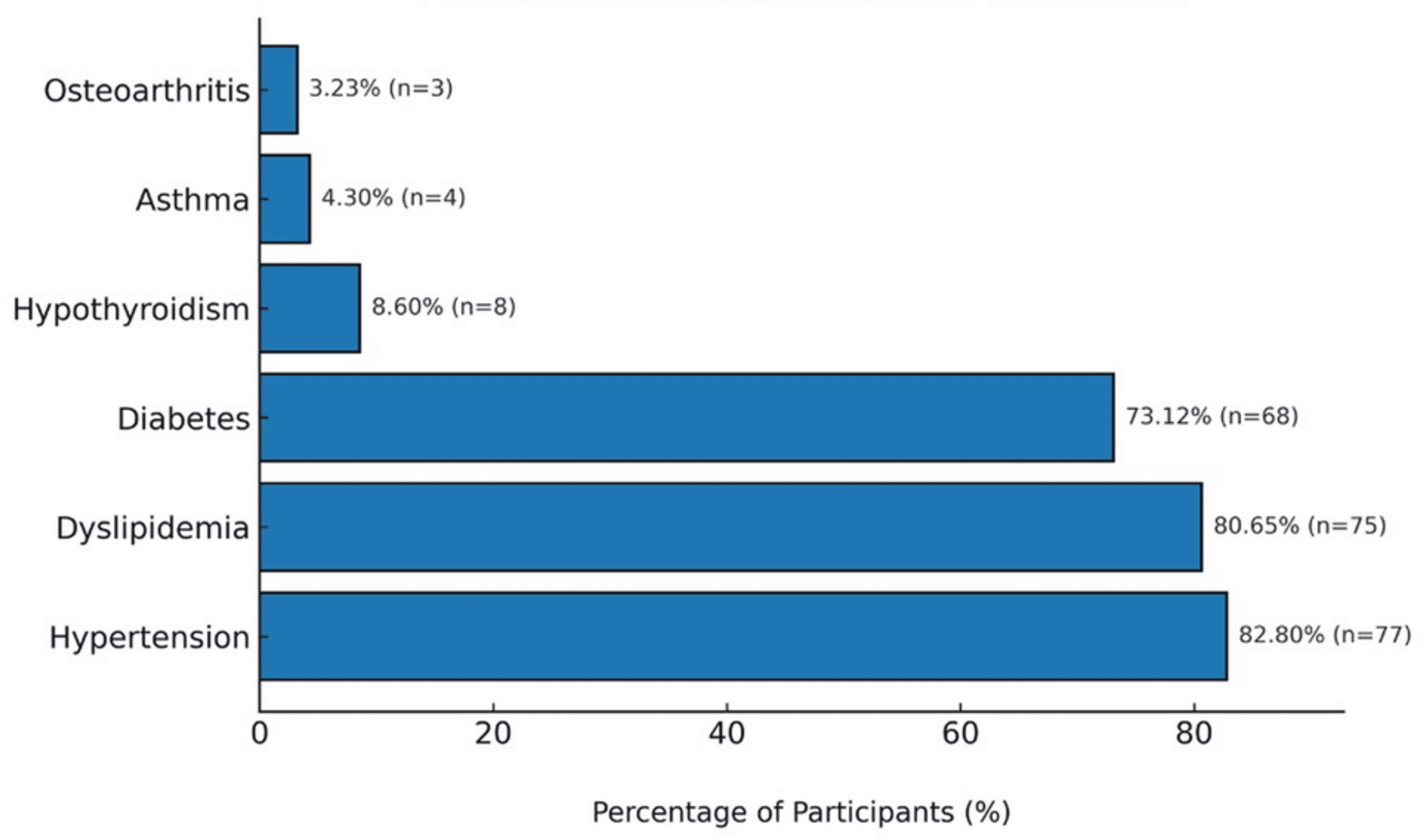
Prevalence of Chronic Medical Conditions Among Participants (N = 93)

As shown in Figure 3, the distribution of comorbidities demonstrated that hypertension, dyslipidemia, and diabetes were the most common conditions, whereas hypothyroidism, asthma, and osteoarthritis were less frequent.

**Figure 4:**
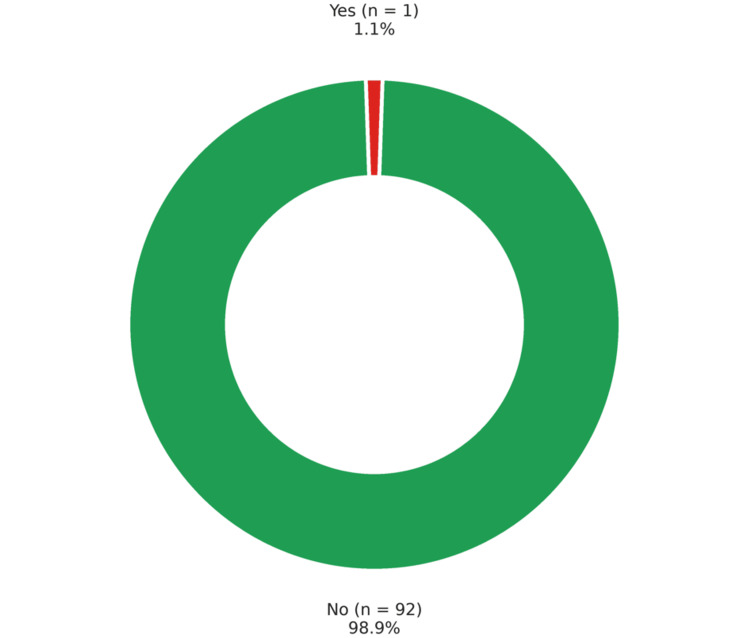
Prevalence of Clinically Diagnosed Leg Length Discrepancy (LLD) Among Participants (N = 93)

Figure 4 shows that clinically diagnosed LLD was uncommon: 92 (98.90%) of participants had no LLD, with only one (1.10%) participant identified as having an LLD of 2.7 cm.

**Figure 5:**
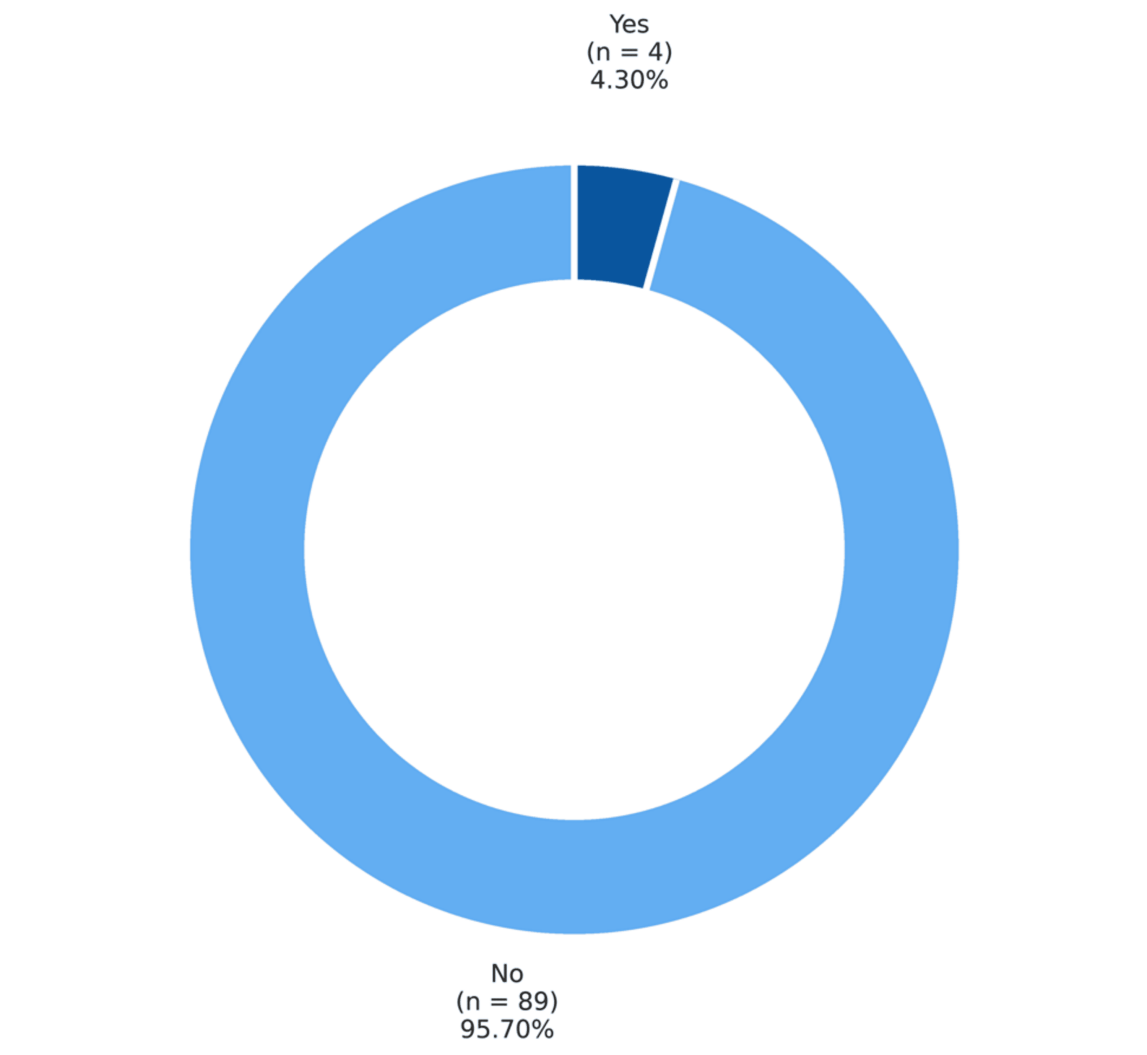
Prevalence of Structural Foot Abnormalities Among Participants (N = 93)

Structural foot abnormalities were reported by only a small minority of participants (4 (4.30%)), as shown in Figure 5; among those affected, the reported abnormalities were pes planus.

Table 2 shows that high-heeled shoes were the most commonly worn footwear during daily activities (29 (31.18%)), followed by flat sandals/slippers (26 (27.96%)) and athletic shoes/sneakers (21 (22.58%)). Smaller proportions used custom orthotic footwear (9 (9.68%)) or occupational/safety footwear (8 (8.60%)).

**Table 2:** Distribution of Most Commonly Worn Footwear Types During Daily Activities Among Participants (N = 93) Flat sandals refer to non-supportive open footwear (e.g., slippers). Athletic shoes include cushioned sneakers or walking shoes with arch support. High-heeled shoes are defined as footwear with a heel height ≥ 2 inches. Occupational/safety footwear refers to work boots or protective shoes. Custom orthotic footwear is medically prescribed supportive shoes.

Footwear type	n (%)
High-heeled shoes	29 (31.18%)
Flat sandals or slippers	26 (27.96%)
Athletic shoes or sneakers	21 (22.58%)
Custom orthotic footwear	9 (9.68%)
Occupational or safety footwear	8 (8.60%)
Total	93 (100%)

**Figure 6:**
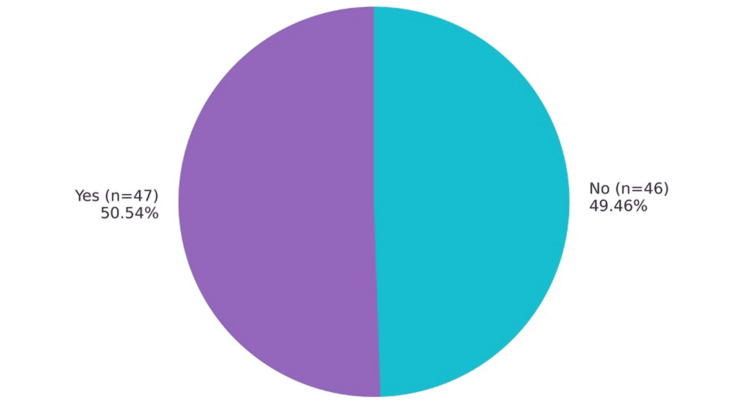
Prevalence of Calf or Intrinsic Foot Muscle Tightness Among Participants (N = 93)

Alongside these findings, Figure 6 demonstrates that calf or intrinsic foot muscle tightness was present in 47 (50.54%) of participants.

**Figure 7:**
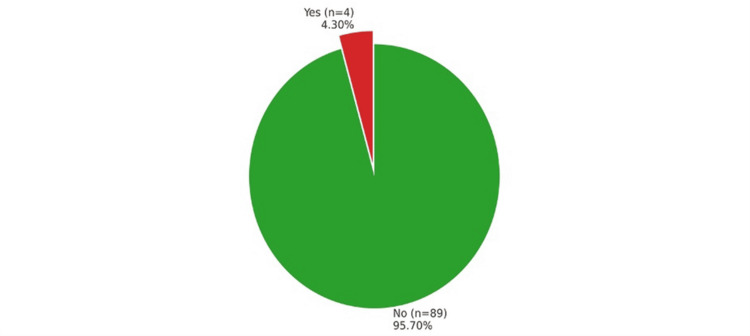
Prevalence of Leg, Ankle, or Foot Trauma or Surgery Prior to Plantar Fasciitis Symptom Onset (N = 93)

Figure 7 shows that only a small minority of participants (4 (4.30%)) reported a history of leg, ankle, or foot trauma or surgery prior to symptom onset. Of these, three (75.00%) cases involved ankle sprains managed conservatively, and one (25.00%) participant had undergone bunionectomy.

**Figure 8:**
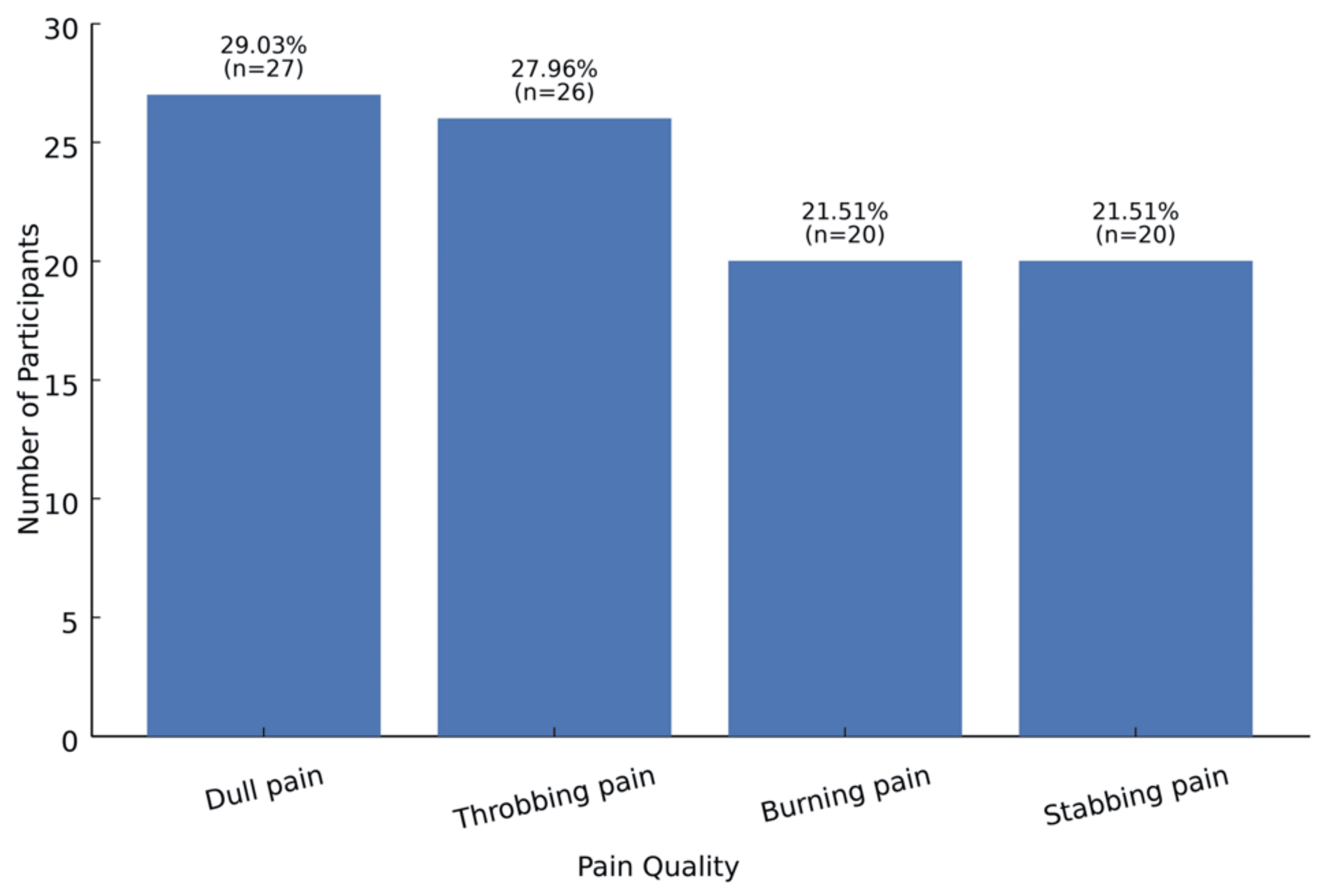
Distribution of Foot/Heel Pain Quality Among Participants (N = 93)

Pain quality encompassed several classic descriptors, with dull pain (27 (29.03%)) and throbbing pain (26 (27.96%)) occurring most frequently, while burning and stabbing sensations were each reported by 20 (21.51%) participants, as illustrated in Figure 8.

**Figure 9:**
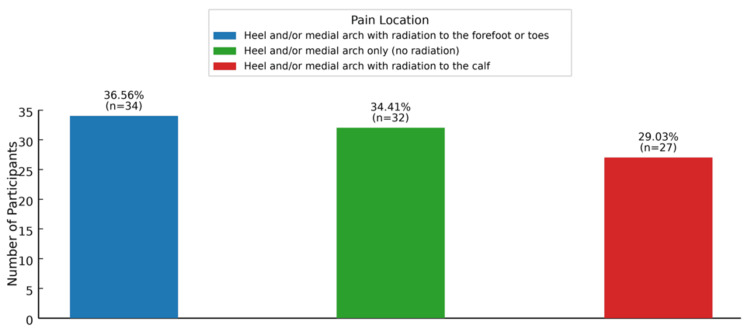
Primary Location of Plantar Fasciitis Pain Among Participants (N = 93)

**Figure 10:**
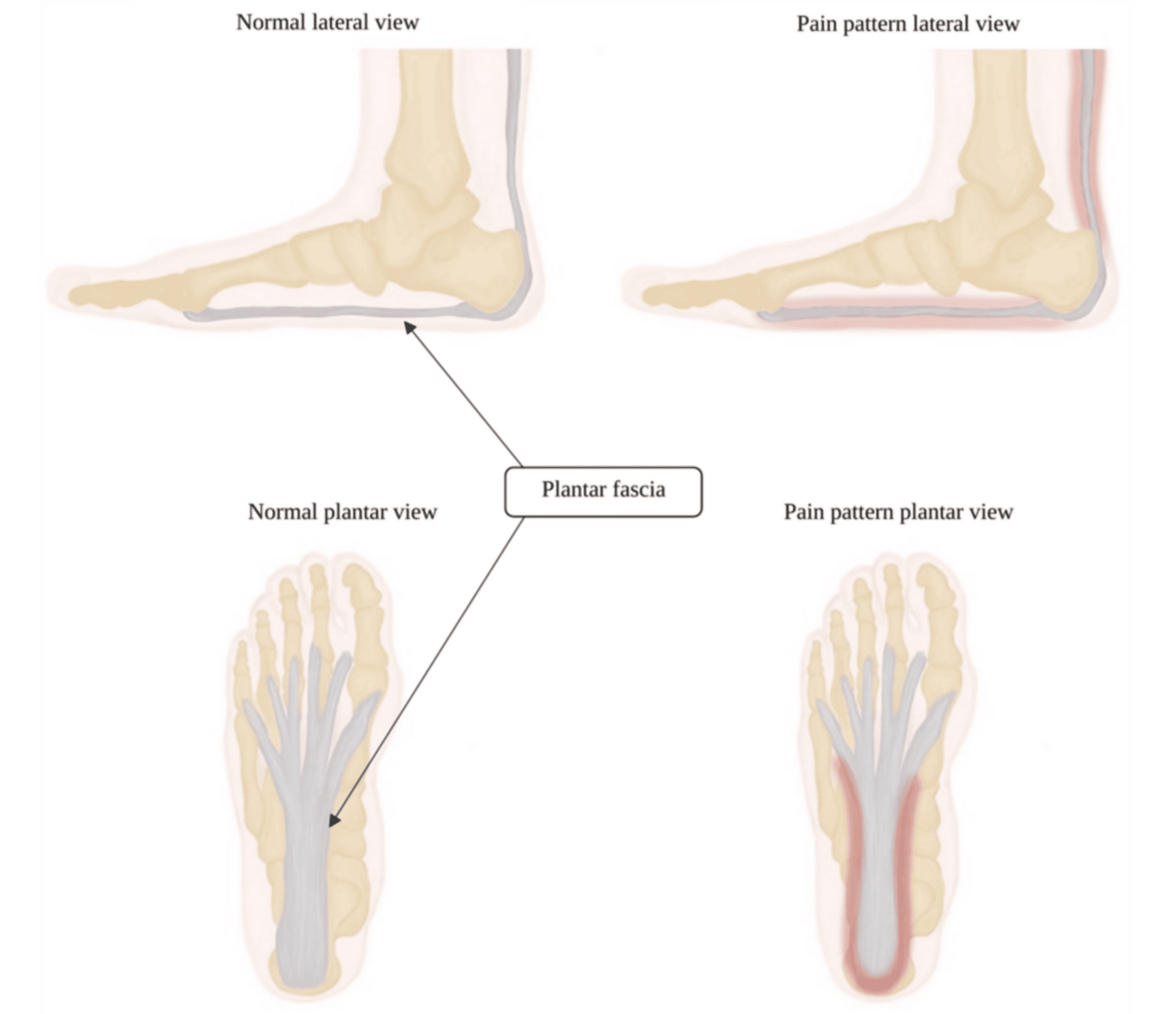
Visual Representation of Plantar Fasciitis Pain Location and Radiation Pattern Red shadowing illustrates the commonly reported pain distribution around the plantar fascia, with radiation along the plantar aspect of the foot and proximal extension toward the Achilles tendon region. The figure demonstrates the typical lateral and plantar views of pain localization and radiation patterns associated with plantar fasciitis. This figure is an original illustration by Ghada F. AlSwaji, created using Sketchbook (www.sketchbook.com).

The primary pain location was most commonly described as heel and/or medial arch pain with radiation to the forefoot or toes, 34 (36.56%), followed by heel and/or medial arch pain without radiation, 32 (34.41%). A smaller proportion described heel and/or medial arch pain radiating to the calf, 27 (29.03%), as shown in Figures 9, 10.

As shown in Table 3, plantar fasciitis symptoms were overwhelmingly insidious in onset, with nearly all participants reporting a gradual onset of pain (90 (96.77%)), while only a small minority described a sudden onset (3 (3.23%)). Regarding diurnal variation, pain patterns were heterogeneous, but the most commonly reported “worst time” was morning pain upon taking the first steps (22 (23.66%)). Other frequently reported peak periods included pain after prolonged standing or walking (≥30 minutes) (20 (21.51%)), pain after prolonged sitting (≥30 minutes) (18 (19.35%)), and pain in the late evening or at night (18 (19.35%)). Notably, a subset of participants reported no significant fluctuation throughout the day (15 (16.13%)). In terms of laterality, foot involvement was predominantly unilateral and right-sided, with the right foot affected in the majority (79 (84.95%)), compared with the left foot (10 (10.75%)) and bilateral involvement (4 (4.30%)).

**Table 3:** Distribution of Pain Onset, Worst Time of Day for Pain, and Foot Involvement Among Participants (N = 93)

Plantar fasciitis pain	Symptom characteristic	n (%)
Initial onset of pain	Gradual onset	90 (96.77%)
	Sudden onset	3 (3.23%)
Total		93 (100%)
Worst time of day for pain	Worse in the morning (upon first steps)	22 (23.66%)
	Worse after prolonged standing or walking (≥30 minutes)	20 (21.51%)
	Worse after prolonged sitting (≥30 minutes)	18 (19.35%)
	Worse in the late evening or at night	18 (19.35%)
	The pain does not vary during the day (no major fluctuation)	15 (16.13%)
Total		93 (100%)
Affected foot	Right foot	79 (84.95%)
	Left foot	10 (10.75%)
	Both feet	4 (4.30%)
Total		93 (100%)

**Figure 11:**
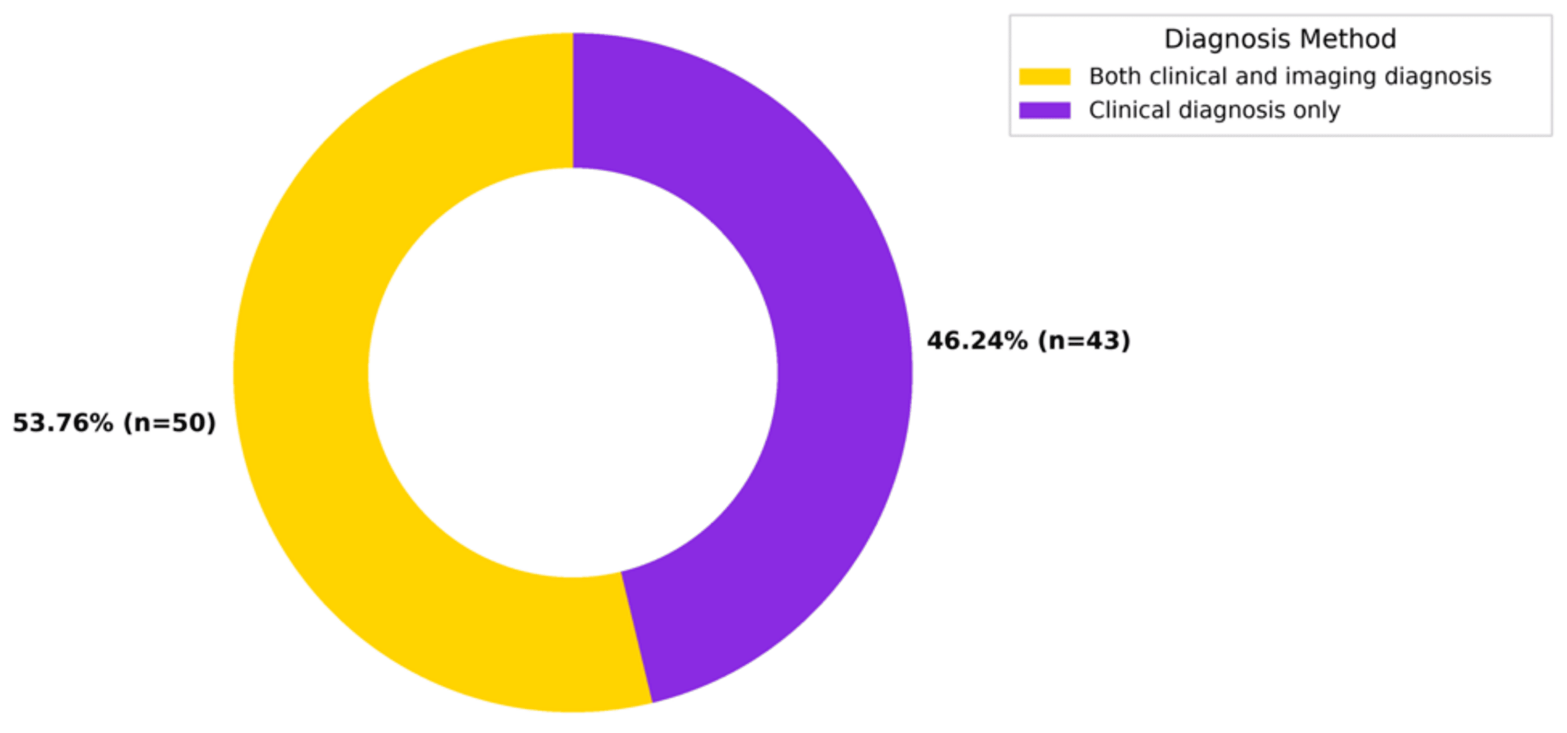
How Plantar Fasciitis Was Diagnosed Among Participants (N = 93)

The diagnosis of plantar fasciitis among participants was made through two main pathways. Slightly more than half of the cases were confirmed using a combined approach of clinical assessment plus imaging (50 (53.76%)), while the remainder were diagnosed based on clinical evaluation alone (43 (46.24%)), as illustrated in Figure 11.

**Figure 12:**
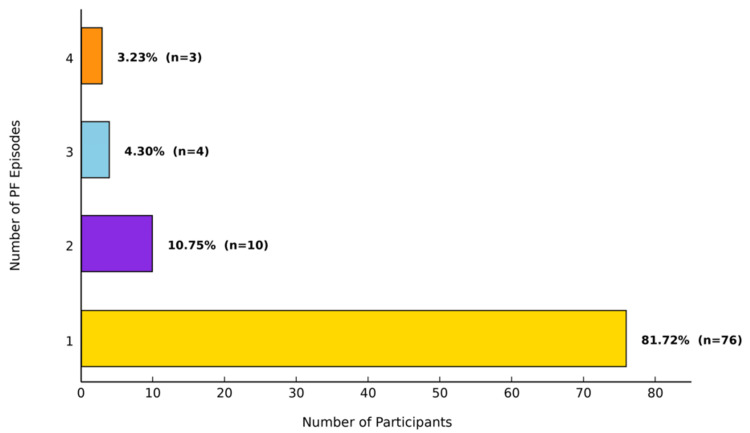
Frequency of Plantar Fasciitis (PF) Episodes Over a Two-Year Period Among Participants (N = 93) A recurrent episode is defined as a new occurrence of PF following a pain-free interval of at least three consecutive months. A single episode refers to any episode of PF without a ≥3-month pain-free interval, and it may be classified as acute (<6 weeks), subacute (6-12 weeks), or chronic (>12 weeks) in duration.

Most participants reported a single plantar fasciitis episode over the previous two years, accounting for 76 (81.72%). In contrast, recurrent episodes were less common: 10 (10.75%) experienced two episodes, four (4.30%) reported three episodes, and only three (3.23%) had four episodes, as represented in Figure 12.

**Figure 13:**
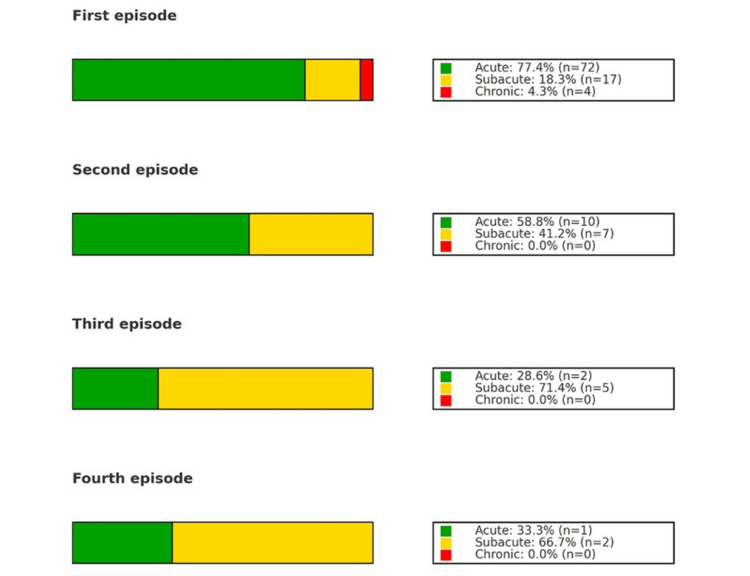
Duration of Symptoms in First and Recurrent Episodes of Plantar Fasciitis Among Participants A recurrent episode is defined as a new occurrence of plantar fasciitis following a pain-free interval of at least three consecutive months. A single episode refers to any episode of plantar fasciitis without a ≥3-month pain-free interval, and it may be classified as acute (<6 weeks), subacute (6-12 weeks), or chronic (>12 weeks) in duration.

As presented in Figure 13, symptom duration in the first plantar fasciitis episode was predominantly acute, with 72 (77.40%) reporting an acute course. A smaller proportion experienced a subacute duration (17 (18.30%)), while only four (4.30%) progressed to a chronic pattern during the initial episode.

Among participants who experienced recurrent episodes, the symptom course remained largely non-chronic, but the distribution shifted toward longer subacute durations with recurrence. In the second episode, symptoms were mainly acute (10 (58.80%)), with a substantial subacute component (7 (41.20%)), and no chronic cases were reported. This trend became more pronounced in the third episode, where subacute symptoms predominated (5 (71.40%)) compared with acute symptoms (2 (28.60%)), again with no chronic presentations. Similarly, during the fourth episode, most cases were subacute (2 (66.70%)) rather than acute (1 (33.30%)), with 0% chronicity. Overall, our participants suggest that while most episodes, especially the first, present acutely, recurrent episodes tend to be associated with a greater proportion of subacute symptom duration, indicating a tendency toward more prolonged recovery periods rather than true chronic persistence.

**Figure 14:**
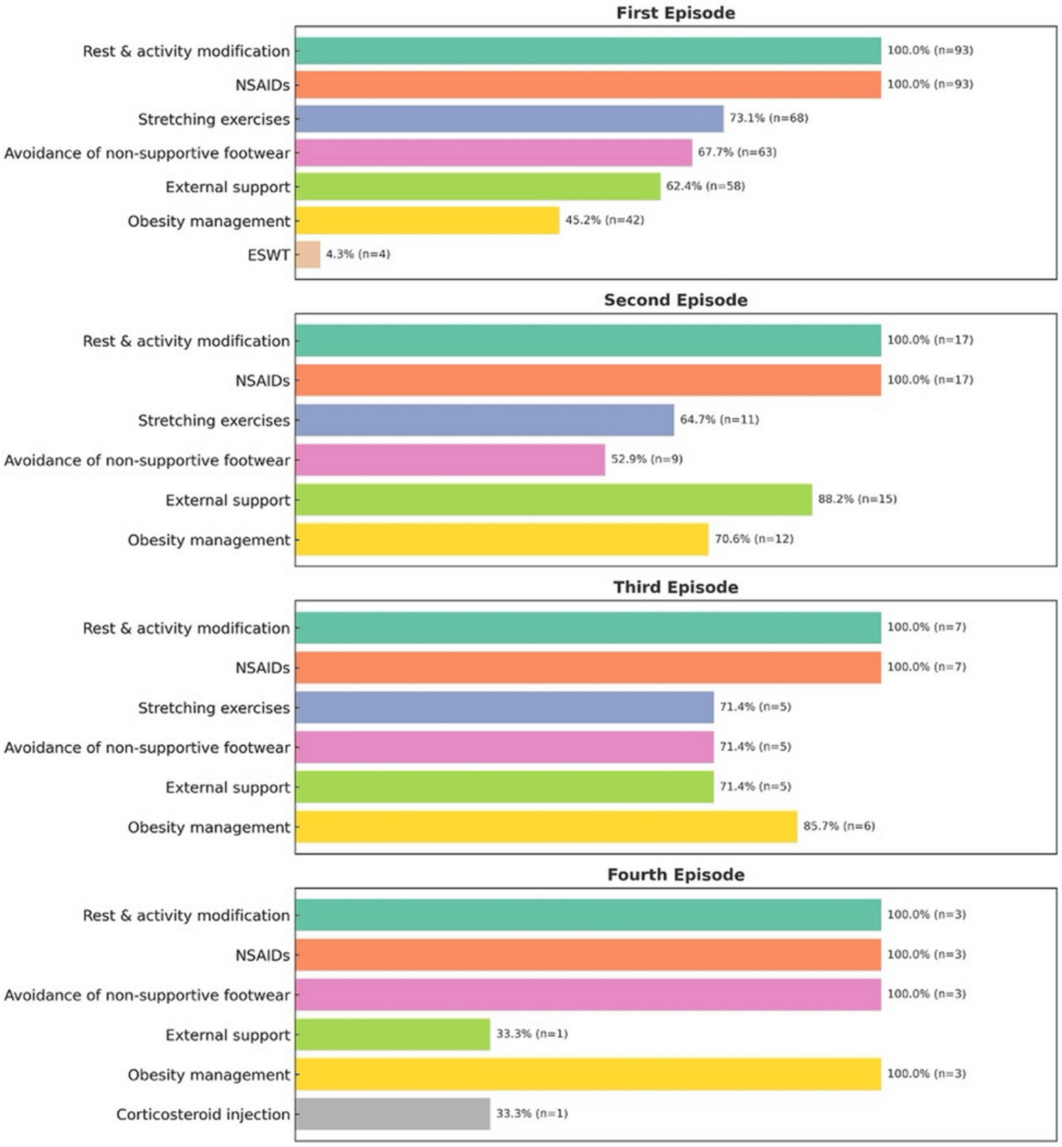
Management Strategies Used Across First and Recurrent Episodes of Plantar Fasciitis Rest/activity modification: reducing activities that aggravate symptoms; NSAIDs (non-steroidal anti-inflammatory drugs): anti-inflammatory medications used for pain relief; stretching/strengthening exercises: exercises targeting the plantar fascia, Achilles tendon, and calf muscles; avoidance of non-supportive footwear: avoiding flat or unsupportive shoes; external support: taping, heel cups, insoles, or orthotics for arch support; obesity management: weight-related interventions to reduce plantar fascia load; ESWT (extracorporeal shockwave therapy): shockwave therapy used to stimulate tissue healing; corticosteroid injection: local anti-inflammatory injection for plantar fasciitis symptoms

As demonstrated in Figure 14, management of plantar fasciitis across episodes remained predominantly conservative, with rest/activity modification and NSAIDs used universally in every episode: first episode, 93 (100.0%); second episode, 17 (100.0%); third episode, 7 (100.0%); and fourth episode, 3 (100.0%). During the first episode, additional supportive measures were also commonly adopted, including stretching exercises (68 (73.1%)), avoidance of non-supportive footwear (63 (67.7%)), and external support (58 (62.4%)), while obesity management was reported by less than half (42 (45.2%)). Only a small minority received ESWT (4 (4.3%)) in the first episode.

With recurrence, the pattern suggests greater reliance on supportive interventions and risk-factor modification. In the second episode, external support was used by most participants (15 (88.2%)), and obesity management increased (12 (70.6%)), rising further in the third episode (6 (85.7%)) and reaching 3 (100.0%) by the fourth episode. Notably, by the fourth episode, all participants reported avoidance of non-supportive footwear (3 (100.0%)), and a minority progressed to corticosteroid injection (1 (33.3%)), indicating selective escalation beyond standard conservative care.

**Figure 15:**
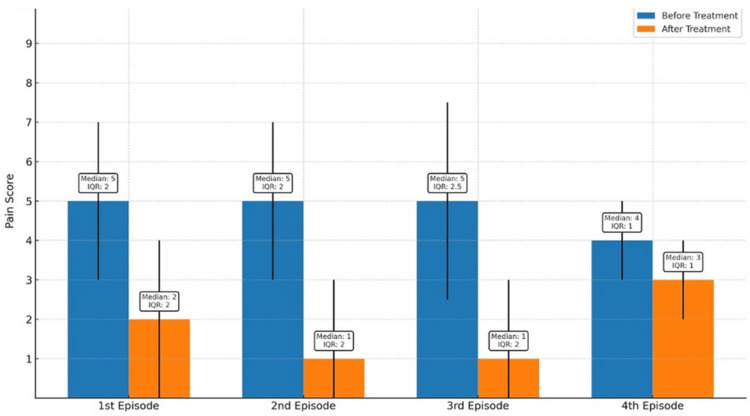
Comparison of Pain Severity Before and After Treatment Across Plantar Fasciitis Episodes This figure compares the severity of plantar fasciitis pain before and after treatment across the first, second, third, and fourth episodes. Bars represent the median pain score on a 0-10 Numeric Rating Scale, while the vertical lines indicate the interquartile range (IQR), reflecting the variability of pain responses among participants.

As shown in Figure 15, pain severity demonstrated a consistent improvement after treatment across plantar fasciitis episodes, as reflected by reductions in the median pain score and, in most episodes, a narrowing of symptom burden. In the first episode, the median pain score decreased from 5.00 (IQR = 2.00) before treatment to 2.00 (IQR = 2.00) after treatment. In the second episode, pain improved further, dropping from 5.00 (IQR = 2.00) to 1.00 (IQR = 2.00) after treatment. A similar pattern was observed in the third episode, where pain declined from 5.00 (IQR = 2.50) before treatment to 1.00 (IQR = 2.00) after treatment. In contrast, the fourth episode showed a more modest reduction, from 4.00 (IQR = 1.00) to 3.00 (IQR = 1.00).

**Figure 16:**
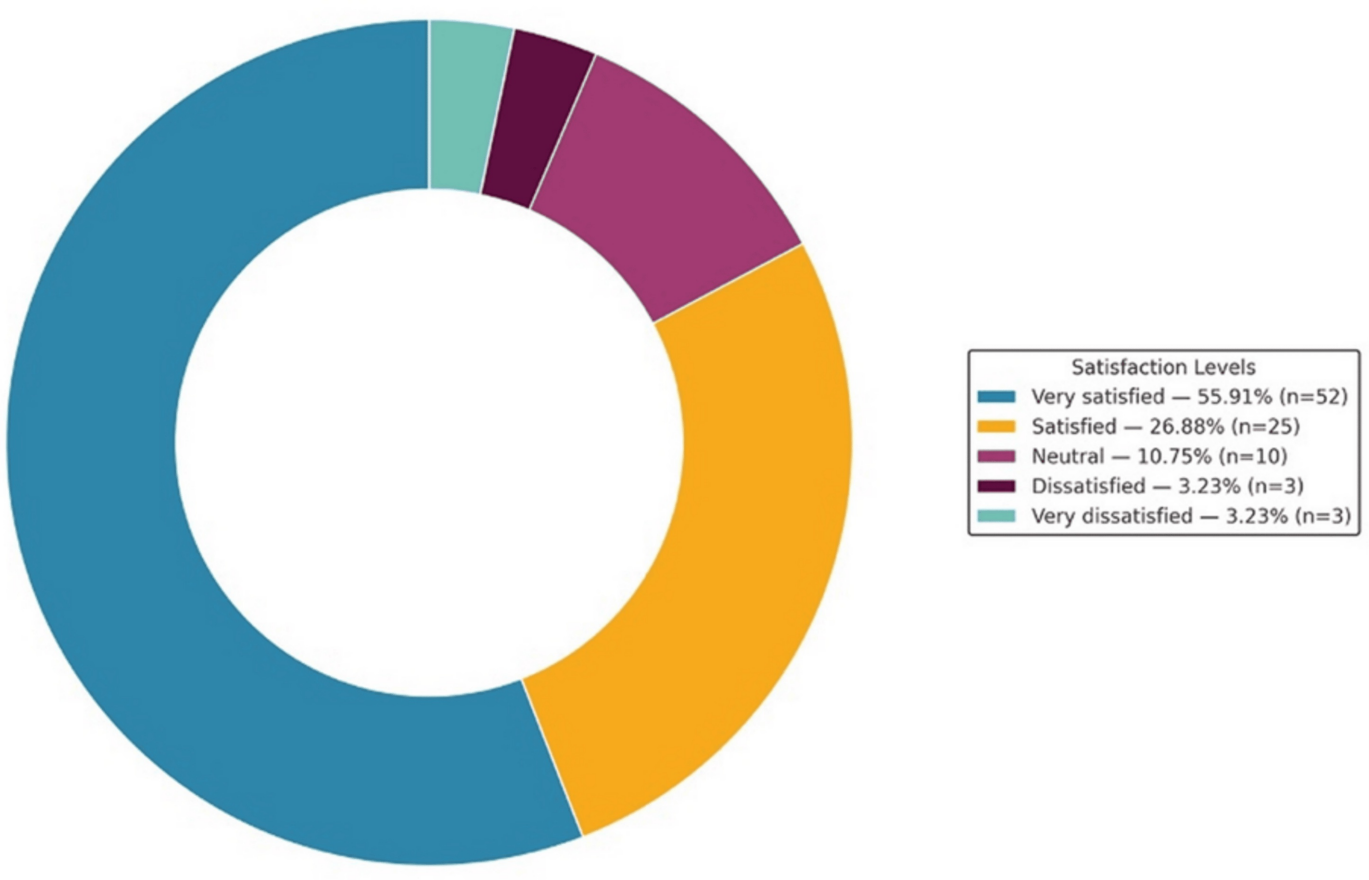
Overall Treatment Satisfaction Among Participants With Plantar Fasciitis (N = 93)

Overall treatment satisfaction was largely favorable among participants with plantar fasciitis. More than half reported being very satisfied (52 (55.91%)), and an additional quarter were satisfied (25 (26.88%)), indicating a high overall acceptance of the management received. A smaller proportion expressed neutral satisfaction (10 (10.75%)), while only a minority reported dissatisfaction, including dissatisfied (3 (3.23%)) and very dissatisfied (3 (3.23%)). Collectively, the majority fell within the satisfied spectrum, supporting an overall positive perceived treatment response, as illustrated in Figure 16.

As shown in Table 4, obesity was significantly associated with recurrence of plantar fasciitis (χ² = 4.25, df = 1, p = 0.039). Recurrence was reported in 12 (28.57%) obese participants compared with five (9.80%) non-obese participants. In contrast, occupational activity level and physical activity level based on WHO recommendations were not significantly associated with recurrence (p = 0.4504 and p = 0.5865, respectively).

**Table 4:** Association of Selected Clinical and Lifestyle Factors With Recurrence of Plantar Fasciitis (N = 93) Recurrence was compared across obesity status, occupational activity level, and physical activity level using the Chi-squared test of independence. The Chi-squared statistic (χ²), degrees of freedom (df), and corresponding p-value are presented for each variable. A p-value < 0.05 was considered statistically significant. Physical activity level was classified according to World Health Organization (WHO) recommendations.

Variable	Category	Recurrence: no, n (%)	Recurrence: yes, n (%)	Total, n (%)	Statistical test	Test statistic (χ²)	df	p-value
Obesity status	Non-obese	46 (90.20%)	5 (9.80%)	51 (54.84%)	Chi-squared test	4.25	1	0.039
	Obese	30 (71.43%)	12 (28.57%)	42 (45.16%)				
	Total	76 (81.72%)	17 (18.28%)	93 (100%)				
Occupational activity level	Active	18 (90.00%)	2 (10.00%)	20 (21.51%)	Chi-squared test	0.57	1	0.4504
	Sedentary	58 (79.45%)	15 (20.55%)	73 (78.49%)				
	Total	76 (81.72%)	17 (18.28%)	93 (100%)				
Physical activity level based on WHO recommendations	Does not meet WHO recommendations	55 (79.71%)	14 (20.29%)	69 (74.19%)	Chi-squared test	0.30	1	0.5865
	Meets WHO recommendations	21 (87.50%)	3 (12.50%)	24 (25.81%)				
	Total	76 (81.72%)	17 (18.28%)	93 (100%)				

## Discussion

In this single-center cohort of 93 adults with plantar fasciitis, the main findings were that most participants were middle-aged adults, with a modest female predominance, high rates of sedentary occupation and physical inactivity, frequent calf or intrinsic foot muscle tightness, and predominantly gradual-onset heel or medial arch pain. Most cases were single episodes over the prior two years, and management was mainly conservative across episodes. Patient-reported pain scores generally improved after treatment, and obesity was significantly associated with recurrence.

Initial management of plantar fasciitis improves symptoms in approximately 80% of patients within 12 months and usually includes rest, avoidance of aggravating factors, pressure reduction using heel inserts, and management of contributing conditions [1]. However, the evidence for how much these measures accelerate recovery remains uncertain [3,4]. Reducing training load has been associated with a lower risk of lower-limb soft tissue injuries, including plantar fasciitis, in a meta-analysis of 8,806 runners [5]. Conservative treatment options such as stretching exercises and shoe inserts have also shown variable benefit; for example, one study of 236 patients reported higher symptom improvement with silicone heel inserts plus stretching than with stretching alone [6].

Other interventions may be considered when symptoms persist. Glucocorticoid injections appear to provide modest short-term pain relief, based on a 2017 Cochrane review [7], and a 2019 study reported greater short-term improvement when injections were used compared with exercise alone, with better six-month outcomes when injections were combined with exercise [8]. The benefit of stretching alone remains uncertain, although targeted plantar fascia stretching may provide greater short-term pain relief than general stretching in some patients [9]. NSAIDs may improve pain and disability when used alongside conservative treatment, although one randomized placebo-controlled study found no statistically significant difference [10].

Plantar fasciitis may become subacute, 6-12 weeks, or chronic, more than 12 weeks, and refractory cases may require ESWT or surgery [2]. A meta-analysis of seven randomized controlled trials found ESWT to be safe and effective for chronic plantar fasciitis resistant to conservative management, with benefits lasting up to 12 months [11]. Surgical approaches, including endoscopic plantar fasciotomy, have also shown pain and functional improvement in chronic cases, with some evidence suggesting faster recovery and fewer complications using a superficial fascial approach [12].

The demographic pattern observed in our cohort was generally consistent with previously described epidemiologic patterns. The sample was predominantly aged 41-70 years, with the 51-60 age group representing the largest proportion, 29 (31.18%), and there was a modest female predominance, 51 (54.84%). In line with the patterns described in Plantar fasciitis by Trojian and Tucker and by Buchanan et al., the observed age distribution reflects the typical midlife peak, approximately 40-60 years, and consistently higher occurrence reported among women [1,13]. In a large Dutch primary care cohort, a higher incidence was reported in women, 4.64 per 1,000 patient-years, compared with men, 2.98 per 1,000 patient-years, supporting the female predominance observed in clinical settings [14].

The clinical profile in our cohort was characterized by high proportions of sedentary occupation, 73 (78.49%), and physical inactivity, 69 (74.19%). While plantar fasciitis is frequently associated with occupations involving prolonged standing or walking, it can also affect sedentary individuals and is described as multifactorial in origin. A substantial minority in our cohort reported smoking or vaping, 34 (36.56%), with a mean exposure of 13 pack-years. In the long-term follow-up cohort reported by Hansen et al. [15], smoking status was not significantly associated with long-term symptom resolution, indicating no measurable effect of smoking on prognosis.

From a biomechanical perspective, calf or intrinsic foot muscle tightness was common, 47 (50.54%). This aligns with widely cited clinical risk factors that include intrinsic foot or calf muscle tightness and altered foot biomechanics. In our cohort, structural contributors were infrequently documented, including clinically diagnosed LLD, 1 (1.10%), while 92 (98.90%) had no LLD, and structural foot abnormalities, 4 (4.30%), all reported as pes planus. Footwear patterns showed that high-heeled shoes were the most commonly reported daily footwear, 29 (31.18%), followed by flat sandals or slippers, 26 (27.96%), and athletic shoes or sneakers, 21 (22.58%). The role of footwear has been emphasized in a cross-sectional study conducted by Umar et al. in Pakistan [16]; most of the 101 patients with plantar fasciitis were wearing inappropriate footwear, 83.20%, while only 16.80% wore recommended footwear. Inappropriate footwear was associated with more severe heel pain, p = 0.013. In a computational biomechanics modeling study by Wang et al. [17], combined musculoskeletal modeling and finite-element analysis showed that plantar fascia strain increased progressively as heel height rose from 3 to 7 cm during high-heel walking. The study also showed that high-heel designs with narrow heel support may increase the risk of developing plantar fasciitis.

Clinically, plantar fasciitis pain in our cohort was overwhelmingly gradual in onset, 90 (96.77%). Morning pain on first steps was the most frequently reported peak time, 22 (23.66%), with other common peaks after prolonged standing or walking, 20 (21.51%), after prolonged sitting, 18 (19.35%), and late evening or night, 18 (19.35%). This pattern is consistent with classical descriptions in which pain is typically worse with the first steps in the morning or after periods of rest and may worsen again later in the day [13,14]. Pain most often involved heel or medial arch pain with radiation to the forefoot or toes, 34 (36.56%), followed by non-radiating heel or medial arch pain, 32 (34.41%), and radiation to the calf, 27 (29.03%). Laterality was predominantly right-sided, 79 (84.95%). In general descriptions, plantar fasciitis can present bilaterally in approximately one-third of cases [14], indicating that laterality patterns may vary between cohorts depending on the population. The marked right-sided predominance observed in our cohort may reflect limb dominance, asymmetric loading patterns, occupational or daily activity habits, or differences in weight distribution during standing and walking. However, because foot dominance and detailed gait or biomechanical assessment were not evaluated in this study, this finding should be interpreted cautiously. Future studies may further explore whether limb dominance, gait mechanics, or occupational posture contribute to laterality patterns in plantar fasciitis.

Diagnostic pathway and episode pattern

Slightly more than half of the cases in our cohort were diagnosed using clinical assessment plus imaging, 50 (53.76%), while the remainder were diagnosed clinically without imaging, 43 (46.24%). The imaging modalities used were ultrasound and foot X-ray. Clinical diagnosis is commonly emphasized in primary care guidance, with imaging generally reserved for atypical presentations or persistent symptoms not responding to therapy. When imaging is performed, ultrasound may show plantar fascia thickening, hypoechogenicity, perifascial fluid, or loss of the normal fibrillar pattern, while X-ray may show a calcaneal heel spur or help exclude alternative causes of heel pain. In contrast to our relatively higher imaging use, Rasenberg et al. [14] reported that diagnostic investigations were ordered at the first consultation in 8.60% of patients, most commonly a foot X-ray in 7.70%, with ultrasound used in 0.90%.

Most participants in our cohort reported a single episode over the prior two years, 76 (81.72%), with fewer reporting two, 10 (10.75%); three, 4 (4.30%); or four episodes, 3 (3.23%). Symptom duration during the first episode was predominantly acute, 72 (77.40%), with smaller proportions subacute, 17 (18.30%), and chronic, 4 (4.30%). Across recurrent episodes, there was a visible shift toward subacute courses without chronicity being reported in subsequent episodes, second to fourth. However, long-term follow-up data suggest that symptom persistence may be substantial in severe cases. In a long-term follow-up study by Hansen et al. [15], the estimated probability of still having plantar fasciitis remained high at 80.50% at one year and 50.00% at five years, with persistence continuing to be observed at 10 and 15 years.

Treatment patterns and patient-reported outcomes

Management was mainly conservative across episodes in our cohort. Rest or activity modification and NSAIDs were used in every reported episode: first episode, 93 (100%); second episode, 17 (100%); third episode, 7 (100%); and fourth episode, 3 (100%). During the first episode, stretching exercises, 68 (73.10%); avoidance of non-supportive footwear, 63 (67.70%); and external support, 58 (62.40%), were common, whereas obesity management was reported by less than half, 42 (45.20%), and ESWT was used infrequently, 4 (4.30%). These conservative strategies are consistent with American Academy of Family Physicians (AAFP) clinical guidance, which supports non-operative management as the initial approach for most patients with plantar fasciitis and notes that approximately 80% of patients improve within 12 months with non-operative therapy [1]. However, in Saudi Arabia, there is no single universally accepted consensus practice approach for plantar fasciitis management, particularly regarding the selection and sequencing of treatment modalities. Contemporary physical therapy guidelines also emphasize structured conservative approaches, including stretching and other non-operative modalities [18].

Across episodes, patient-reported pain severity improved after treatment. The median pain score decreased from 5.00, IQR = 2.00, to 2.00, IQR = 2.00, in the first episode; from 5.00, IQR = 2.00, to 1.00, IQR = 2.00, in the second episode; and from 5.00, IQR = 2.50, to 1.00, IQR = 2.00, in the third episode. In the fourth episode, improvement was more modest, from 4.00, IQR = 1.00, to 3.00, IQR = 1.00. These patterns align with the high overall satisfaction reported: very satisfied, 52 (55.91%), and satisfied, 25 (26.88%), predominated, with smaller proportions neutral, 10 (10.75%); dissatisfied, 3 (3.23%); and very dissatisfied, 3 (3.23%).

Regarding ESWT, although it was infrequently used in our cohort, published evidence from a meta-analysis of randomized controlled trials supports its use in chronic plantar fasciitis resistant to conservative management. The meta-analysis included nine studies involving 935 patients and found that ESWT had higher improvement rates than placebo. However, the authors noted that no firm conclusion could be drawn regarding the effectiveness of general ESWT and radial shockwave therapy because of heterogeneity, while focused shockwave therapy appeared to provide pain relief in chronic plantar fasciitis [11]. In addition, Malliaropoulos et al. [19] reported results from a retrospective study of radial ESWT including 68 patients, 78 affected heels, treated between 2006 and 2013. The authors observed a marked reduction in the mean visual analog scale pain score, from 6.9 before treatment to 0.9 at the one-year follow-up. Using a criterion of more than 60% pain reduction to define treatment success, they reported success rates of 19% at one month, 70% at three months, and 98% at one year, with a one-year recurrence rate of 8%.

Recurrence and associated factors

A key finding in our cohort was the statistically significant association between obesity and recurrence, χ² = 4.25, df = 1, p = 0.039. Recurrence occurred in 12 (28.57%) obese participants versus five (9.80%) non-obese participants. This finding is consistent with broader evidence identifying elevated BMI as a clinically important factor in plantar fasciopathy. In a systematic review and meta-analysis by van Leeuwen et al. [20], a BMI greater than 27 was significantly associated with plantar fasciopathy, with an odds ratio of 3.70, 95% confidence interval 2.93-5.62. The authors further noted that, across the pooled variables assessed, elevated BMI was the only clinical factor that remained statistically significant. Additionally, in a matched case-control study by Irving et al. [21], obesity, BMI ≥ 30, was associated with higher odds of chronic plantar heel pain, odds ratio 2.90, 95% confidence interval 1.40-6.10. A pronated foot posture, Foot Posture Index ≥ 4, was also associated with increased odds, odds ratio 3.70, 95% confidence interval 1.60-8.70.

Clinically, our findings support prioritizing weight-related risk modification as a central component of recurrence prevention, particularly since obesity management increased in later episodes: second episode, 12 (70.60%); third episode, 6 (85.70%); and fourth episode, 3 (100%). This suggests that patients may adopt risk-factor modification more consistently after recurrence.

Strengths and limitations

A key strength of our study is the systematic and comprehensive collection of demographic characteristics, risk factors, and details for each plantar fasciitis episode, including the number of episodes, symptom duration, and treatments used, together with patient-reported outcomes, using a standardized interview framework. This enabled detailed characterization of symptom patterns, management strategies, and perceived treatment response.

Limitations include the retrospective nature of episode and treatment reporting, telephone-based recall, the single-center setting, which may limit generalizability, and treatment variability across individuals, with no standardized protocol, which may influence comparative interpretation across management strategies. Additional limitations include the potential for recall bias due to telephone-based reporting of previous episodes, symptom duration, and treatment use, as well as possible non-response bias because some eligible patients could not be reached or declined participation despite multiple contact attempts. The absence of multivariable analysis is another limitation; because of the modest sample size and low number of recurrence cases, only univariate analyses were performed, and the observed associations should not be interpreted as independent predictors or causal relationships.

Implications for practice

Based on the observed patterns in our study, clinical pathways may benefit from (1) early identification and management of modifiable risks, particularly obesity, given its association with recurrence and its consistent association with plantar fasciopathy in broader evidence; (2) reinforcing core conservative measures, including rest or activity modification, NSAIDs, stretching, external support, and footwear modification, which were widely utilized and aligned with commonly recommended first-line management approaches; and (3) setting expectations that recurrence, when it occurs, may be accompanied by longer subacute symptom durations, 6-12 weeks, rather than progression to chronicity in subsequent episodes, as observed in our study.

## Conclusions

This study highlights that plantar fasciitis in routine clinical practice was commonly managed through conservative approaches, with generally favorable patient-reported pain improvement and satisfaction. These outcomes should be interpreted as occurring after overall conservative management rather than as evidence of the effectiveness of any specific treatment modality. The findings emphasize the importance of early recognition, structured non-operative management, appropriate footwear advice, stretching-based interventions, external support, and individualized follow-up to support recovery and reduce symptom burden.

Recurrence remains an important clinical consideration, particularly in patients with modifiable risk factors. Obesity was associated with recurrence in this cohort; however, given the retrospective design, telephone-based recall, and use of univariate analysis, this finding should be interpreted as an unadjusted association rather than evidence of causality or an independent predictor. Incorporating risk-factor modification, including weight management when appropriate, into routine plantar fasciitis care may help support long-term outcomes and reduce the likelihood of recurrent episodes.

## Appendices

**Table 5:** Study Questionnaire This questionnaire was developed by the study authors for the purpose of this research. Credits: Yasser S. Aljaied, Mohamed Alrubaiaan, Mazin Elsarrag, Bandar ALshehry, Rahaf Almutairi, Ghada F. AlSwaji, Abdulmlk A. Alqasem, and Hamza Enabi

No.	Question/instruction	Response options
1	Age?	☐ 18-30 years ☐ 31-40 years ☐ 41-50 years ☐ 51-60 years ☐ 61-70 years
2	Gender?	☐ Male ☐ Female
3	What is your body mass index (BMI)?	☐ Underweight (BMI < 18.5) ☐ Normal weight (BMI 18.5-24.9) ☐ Overweight (BMI 25-29.9) ☐ Obesity Class I (BMI 30-34.9) ☐ Obesity Class II (BMI 35-39.9) ☐ Obesity Class III/morbid obesity (BMI ≥ 40)
4	Occupation?	☐ Sedentary-primarily seated throughout the day with minimal physical movement (e.g., office-based roles) ☐ Active-requires regular standing or walking, but not intense physical effort (e.g., teaching, retail, and hospitality) ☐ Highly active-involves sustained physical exertion, frequent lifting, or prolonged movement (e.g., construction work, nursing, and military service)
5	Physical activity level (per week) based on WHO recommendation?	☐ Physically inactive-I engage in less than 150 minutes per week of moderate-intensity aerobic activity, or less than 75 minutes per week of vigorous-intensity aerobic activity, and do not perform regular muscle-strengthening activities involving all major muscle groups on at least 2 days/week ☐ Active-I meet the WHO minimum recommendation of 150-300 minutes per week of moderate-intensity aerobic activity, or 75-150 minutes per week of vigorous-intensity aerobic activity, or an equivalent combination, and I perform muscle-strengthening activities involving all major muscle groups on 2 or more days/week ☐ Highly active-I exceed the WHO minimum recommendation: more than 300 minutes per week of moderate-intensity aerobic activity, or more than 150 minutes per week of vigorous-intensity aerobic activity, or an equivalent combination, and I perform muscle-strengthening activities involving all major muscle groups on 2 or more days/week
6	Have you received a clinical diagnosis of leg length discrepancy?	☐ Yes ☐ No. If yes, how much is the leg length discrepancy (in cm)?
7	Has a physician ever diagnosed you with any of the following structural foot abnormalities commonly associated with plantar fasciitis? (Select all that apply.)	☐ Pes planus (flat feet) ☐ Pes cavus (high arches) ☐ Valgus foot ☐ Varus foot ☐ Hallux valgus (bunion) ☐ Tight Achilles tendon (equinus deformity) ☐ Other (please specify): _______________________ ☐ None of the above
8	What type of footwear do you most commonly wear during daily activities? (Not as part of interventional treatment) (Select the option that best represents your primary or most frequently worn footwear.)	☐ Flat sandals or slippers (non-supportive, open footwear without arch support) ☐ Athletic shoes or sneakers (e.g., running shoes, walking shoes with cushioning and arch support) ☐ High-heeled shoes (heels ≥ 2 inches in height) ☐ Occupational or safety footwear (e.g., work boots, steel-toe shoes) ☐ Custom orthotic footwear (medically designed or prescribed supportive shoes) ☐ Other (please specify): _______________________
9	Do you smoke or use vapes?	☐ Yes ☐ No. If yes, how many packs and for how long? Calculate pack-years
10	Do you have any of the following chronic medical conditions? (Check all that apply.)	☐ Diabetes ☐ Hypertension ☐ Dyslipidemia ☐ Hypothyroidism ☐ Hyperthyroidism ☐ Other (please specify): _______________________
11	Have you ever experienced tightness or stiffness in your calf muscles or intrinsic foot muscles?	☐ Yes ☐ No
12	Have you ever had any history of trauma or surgery involving your leg, ankle, or foot before the onset of plantar fasciitis pain?	☐ Yes ☐ No. If yes (please specify): _______________________ (Examples): ☐ Ankle sprain (e.g., inversion injury, lateral ligament strain) ☐ Fracture (e.g., tibia, fibula, calcaneus, metatarsals, or phalanges-including stress fractures) ☐ Heel contusion or blunt trauma (e.g., direct hit, fall from height, or impact to heel pad) ☐ Achilles tendon injury (e.g., strain, tendinopathy, or rupture) ☐ Plantar fascia injury (e.g., partial tear or acute plantar strain) ☐ Muscle strain (e.g., calf strain or foot muscle tear not related to tendon) ☐ Joint dislocation or subluxation (e.g., ankle, midfoot, or toe joints) ☐ Surgical procedure involving the leg, ankle, or foot (e.g., bunionectomy, fracture fixation, or fasciotomy)
13	How would you describe the quality of your foot/heel pain? (Choose only one option.)	☐ Stabbing pain ☐ Dull pain ☐ Burning pain ☐ Throbbing pain ☐ Other
14	Where is your plantar fasciitis pain primarily located? (Choose one option only.)	☐ Heel and/or medial arch only (no radiation) ☐ Heel and/or medial arch with radiation to the forefoot or toes ☐ Heel and/or medial arch with radiation to the calf ☐ Other
15	How would you describe the initial onset of your foot or heel pain? Select the option that best reflects how your symptoms first began.	☐ Gradual onset-pain developed slowly over time ☐ Sudden onset-pain began instantly
16	Is your pain typically worse during specific times of the day?	☐ Yes, worse in the morning (upon first steps) ☐ Yes, worse after prolonged standing or walking (≥30 minutes) ☐ Yes, worse in the late evening or at night ☐ Yes, worse after prolonged sitting (≥30 minutes) ☐ No, my pain does not vary during the day (no major fluctuation) ☐ Other
17	Which foot is affected by your plantar fasciitis symptoms?	☐ Left foot ☐ Right foot ☐ Both feet
18	How was your plantar fasciitis diagnosed?	☐ Clinical diagnosis only (based on symptoms and physical examination) ☐ Both clinical and imaging diagnosis
19	In the past 2 years, how many episodes of plantar fasciitis have you experienced? For this question, a recurrent episode is defined as a new onset of heel pain after being completely pain-free for at least 3 consecutive months. If you have had continuous symptoms over the past 2 years without a pain-free interval of 3 months or more, this should be counted as a single episode, even if the pain fluctuated.	☐ One episode-a single occurrence of plantar heel pain, either acute (<6 weeks), subacute (6-12 weeks), or chronic (>12 weeks) ☐ Two episodes-two distinct episodes, each separated by at least 3 months without pain ☐ Three episodes-three distinct episodes, each separated by at least 3 months without pain ☐ Four episodes-four distinct episodes, each separated by at least 3 months without pain ☐ More than four episodes-more than four distinct episodes, each separated by at least 3 months without pain
20	How long did your first episode of plantar fasciitis symptoms last?	☐ Less than 6 weeks (acute phase) ☐ 6 to 12 weeks (subacute phase) ☐ More than 12 weeks (chronic phase)
21	If you have had more than one episode of plantar fasciitis in the past two years, how long did each episode last (excluding the first)?	Episode number: duration of symptoms. Second episode ☐ Less than 6 weeks ☐ 6-12 weeks ☐ More than 12 weeks. Third episode ☐ Less than 6 weeks ☐ 6-12 weeks ☐ More than 12 weeks. Fourth episode ☐ Less than 6 weeks ☐ 6-12 weeks ☐ More than 12 weeks
22	Which of the following management strategies did you use during your first episode of plantar fasciitis? You may select all that apply.	☐ Rest and activity modification ☐ Avoidance of non-supportive footwear (e.g., flat sandals, ballet slippers, or flip flops) ☐ Use of external support (e.g., foot taping, over the counter or custom orthotic insoles) ☐ Stretching and strengthening exercises specific to the plantar fascia, Achilles tendon, or calf muscles ☐ Obesity management (if advised due to high BMI) ☐ Pain management with NSAIDs (e.g., ibuprofen, diclofenac) ☐ Corticosteroid injection(s) ☐ Botulinum toxin injection(s) ☐ Extracorporeal shockwave therapy (ESWT) ☐ Surgery (e.g., plantar fasciotomy and/or gastrocnemius release) ☐ Other (please specify): ___________________________
23	Which of the following management strategies did you use during your second, third, or fourth episodes of plantar fasciitis? You may select all that apply for each episode.	Second episode ☐ Rest and activity modification ☐ Avoidance of non-supportive footwear (e.g., flat sandals, ballet slippers, or flip flops) ☐ Use of external support (e.g., foot taping, over-the-counter or custom orthotic insoles) ☐ Stretching and strengthening exercises (plantar fascia, Achilles tendon, or calf) ☐ Obesity management (if advised due to high BMI) ☐ Pain management with NSAIDs (e.g., ibuprofen, diclofenac) ☐ Corticosteroid injection(s) ☐ Botulinum toxin injection(s) ☐ Extracorporeal shockwave therapy (ESWT) ☐ Surgery (e.g., plantar fasciotomy and/or gastrocnemius release) ☐ Other (please specify): ___________________________. Third episode ☐ Rest and activity modification ☐ Avoidance of non-supportive footwear ☐ Use of external support ☐ Stretching and strengthening exercises ☐ Obesity management ☐ NSAIDs for pain management ☐ Corticosteroid injection(s) ☐ Botulinum toxin injection(s) ☐ Extracorporeal shockwave therapy (ESWT) ☐ Surgery (e.g., plantar fasciotomy and/or gastrocnemius release) ☐ Other (please specify): ___________________________. Fourth episode ☐ Rest and activity modification ☐ Avoidance of non-supportive footwear ☐ Use of external support ☐ Stretching and strengthening exercises ☐ Obesity management ☐ NSAIDs for pain management ☐ Corticosteroid injection(s) ☐ Botulinum toxin injection(s) ☐ Extracorporeal shockwave therapy (ESWT) ☐ Surgery (e.g., plantar fasciotomy and/or gastrocnemius release) ☐ Other (please specify): ___________________________
24	On a scale from 0 to 10, how severe was your plantar heel pain before receiving treatment during each episode? 0 = No pain, 10 = Worst imaginable pain. Please select one value per episode.	First episode: ☐ 0 ☐ 1 ☐ 2 ☐ 3 ☐ 4 ☐ 5 ☐ 6 ☐ 7 ☐ 8 ☐ 9 ☐ 10. Second episode: ☐ 0 ☐ 1 ☐ 2 ☐ 3 ☐ 4 ☐ 5 ☐ 6 ☐ 7 ☐ 8 ☐ 9 ☐ 10. Third episode: ☐ 0 ☐ 1 ☐ 2 ☐ 3 ☐ 4 ☐ 5 ☐ 6 ☐ 7 ☐ 8 ☐ 9 ☐ 10. Fourth episode: ☐ 0 ☐ 1 ☐ 2 ☐ 3 ☐ 4 ☐ 5 ☐ 6 ☐ 7 ☐ 8 ☐ 9 ☐ 10
25	On a scale from 0 to 10, how severe was your plantar heel pain after receiving treatment during each episode? 0 = No pain, 10 = Worst imaginable pain. Please select one value per episode based on the average pain level after completing treatment.	First episode: ☐ 0 ☐ 1 ☐ 2 ☐ 3 ☐ 4 ☐ 5 ☐ 6 ☐ 7 ☐ 8 ☐ 9 ☐ 10. Second episode: ☐ 0 ☐ 1 ☐ 2 ☐ 3 ☐ 4 ☐ 5 ☐ 6 ☐ 7 ☐ 8 ☐ 9 ☐ 10. Third episode: ☐ 0 ☐ 1 ☐ 2 ☐ 3 ☐ 4 ☐ 5 ☐ 6 ☐ 7 ☐ 8 ☐ 9 ☐ 10. Fourth episode: ☐ 0 ☐ 1 ☐ 2 ☐ 3 ☐ 4 ☐ 5 ☐ 6 ☐ 7 ☐ 8 ☐ 9 ☐ 10
26	Overall, how satisfied were you with the treatment you received for plantar fasciitis? Please consider pain relief, function, comfort, and overall experience.	☐ Very satisfied ☐ Satisfied ☐ Neutral/neither satisfied nor dissatisfied ☐ Dissatisfied ☐ Very dissatisfied

## References

[1] Trojian T, Tucker AK (2019). Plantar fasciitis. Am Fam Physician.

[2] Schneider HP, Baca JM, Carpenter BB, Dayton PD, Fleischer AE, Sachs BD (2018). American College of Foot and Ankle Surgeons clinical consensus statement: diagnosis and treatment of adult acquired infracalcaneal heel pain. J Foot Ankle Surg.

[3] Buchbinder R (2004). Clinical practice. Plantar fasciitis. N Engl J Med.

[4] Crawford F, Thomson CE (2010). Withdrawn. Interventions for treating plantar heel pain. Cochrane Database Syst Rev.

[5] Yeung EW, Yeung SS (2001). A systematic review of interventions to prevent lower limb soft tissue running injuries. Br J Sports Med.

[6] Pfeffer G, Bacchetti P, Deland J (1999). Comparison of custom and prefabricated orthoses in the initial treatment of proximal plantar fasciitis. Foot Ankle Int.

[7] David JA, Sankarapandian V, Christopher PR, Chatterjee A, Macaden AS (2017). Injected corticosteroids for treating plantar heel pain in adults. Cochrane Database Syst Rev.

[8] Johannsen FE, Herzog RB, Malmgaard-Clausen NM, Hoegberget-Kalisz M, Magnusson SP, Kjaer M (2019). Corticosteroid injection is the best treatment in plantar fasciitis if combined with controlled training. Knee Surg Sports Traumatol Arthrosc.

[9] DiGiovanni BF, Nawoczenski DA, Lintal ME, Moore EA, Murray JC, Wilding GE, Baumhauer JF (2003). Tissue-specific plantar fascia-stretching exercise enhances outcomes in patients with chronic heel pain. A prospective, randomized study. J Bone Joint Surg Am.

[10] Donley BG, Moore T, Sferra J, Gozdanovic J, Smith R (2007). The efficacy of oral nonsteroidal anti-inflammatory medication (NSAID) in the treatment of plantar fasciitis: a randomized, prospective, placebo-controlled study. Foot Ankle Int.

[11] Sun J, Gao F, Wang Y, Sun W, Jiang B, Li Z (2017). Extracorporeal shock wave therapy is effective in treating chronic plantar fasciitis: a meta-analysis of RCTs. Medicine (Baltimore).

[12] Çatal B, Keskinbora M, Uysal MA, Şahin M, Gulabi D, Demiralp B (2017). Endoscopic plantar fasciotomy; deep fascial versus superficial fascial approach: a prospective randomized study. J Foot Ankle Surg.

[13] Buchanan BK, Sina RE, Kushner D (2026). Plantar fasciitis. StatPearls [Internet].

[14] Rasenberg N, Bierma-Zeinstra SM, Bindels PJ, van der Lei J, van Middelkoop M (2019). Incidence, prevalence, and management of plantar heel pain: a retrospective cohort study in Dutch primary care. Br J Gen Pract.

[15] Hansen L, Krogh TP, Ellingsen T, Bolvig L, Fredberg U (2018). Long-term prognosis of plantar fasciitis: a 5- to 15-year follow-up study of 174 patients with ultrasound examination. Orthop J Sports Med.

[16] Umar H, Idrees W, Umar W, Khalil A, Rizvi ZA (2022). Impact of routine footwear on foot health: a study on plantar fasciitis. J Family Med Prim Care.

[17] Wang M, Li S, Teo EC, Fekete G, Gu Y (2021). The influence of heel height on strain variation of plantar fascia during high heel shoes walking-combined musculoskeletal modeling and finite element analysis. Front Bioeng Biotechnol.

[18] Koc TA Jr, Bise CG, Neville C, Carreira D, Martin RL, McDonough CM (2023). Heel pain-plantar fasciitis: revision 2023. J Orthop Sports Phys Ther.

[19] Malliaropoulos N, Crate G, Meke M, Korakakis V, Nauck T, Lohrer H, Padhiar N (2016). Success and recurrence rate after radial extracorporeal shock wave therapy for plantar fasciopathy: a retrospective study. Biomed Res Int.

[20] van Leeuwen KD, Rogers J, Winzenberg T, van Middelkoop M (2016). Higher body mass index is associated with plantar fasciopathy/'plantar fasciitis': systematic review and meta-analysis of various clinical and imaging risk factors. Br J Sports Med.

[21] Irving DB, Cook JL, Young MA, Menz HB (2007). Obesity and pronated foot type may increase the risk of chronic plantar heel pain: a matched case-control study. BMC Musculoskelet Disord.

